# Supplementary material for: TastepepAI: An artificial intelligence platform for taste peptide de novo design
Source: PLoS Comput Biol. 2025 Oct 16;21(10):e1013602. doi: 10.1371/journal.pcbi.1013602 (PMC12543283; doi:10.1371/journal.pcbi.1013602)
Supplement: S1 File — (ZIP) [file pcbi.1013602.s006.zip › Supplementary Data 2/TaPep13 HPLC.doc]

**HPLC REPORT**

| Sample: | Fcl-NJP93314-TaPep13 YS-6 | | | Analyzed date: | 2024-9-23 |
| --- | --- | --- | --- | --- | --- |
| Analyst: | LY Reconstitution: 1mg/0.5ml H_2_O | | | | |
| Lot. No.: | P240913-MX1172217 | | | | |
| Column: | 250*4.6mm,ChromCore 120 C18 5um | | | | |
| Solvent A | A: 0.1% Trifluoroacetic Acid in 100% Acetonitrile | | | | |
| Solvent B | B: 0.1% Trifluoroacetic Acid in 100% Water | | | | |
| Gradient: |  | A | B |  | |
|  | 0.0min | 1% | 99% |  | |
|  | 25.0min | 26% | 74% |  | |
|  | 25.1min | 100% | 0% |  | |
|  | 30.0min | Stop | |  | |
| Volume: | 10μl | | |  | |
| Wavelength: | 220nm | | |  | |
| Flow rate: | 1.0ml/min | | |  | |
| ───────────────────────────────────────────────────  Peak No Ret Time Height Area Conc.  ───────────────────────────────────────────────────  1 8.312 367.911 3705.962 0.0511  2 9.377 162.147 843.088 0.0116  3 10.333 1727.400 12655.016 0.1744  4 11.475 4100.579 27635.330 0.3809  5 11.777 17141.125 17530.848 0.2416  6 11.888 542224.188 7134938.000 98.3396  7 11.888 23750.869 56198.398 0.7746  8 13.962 193.284 1058.946 0.0146  9 21.422 202.235 844.116 0.0116  ───────────────────────────────────────────────────  Total 100.00 | | | | | |
